# Dab1 (*Disable Homolog-1*) Reelin Adaptor Protein Is Overexpressed in the Olfactory Bulb at Early Postnatal Stages

**DOI:** 10.1371/journal.pone.0026673

**Published:** 2011-10-25

**Authors:** Eduardo Martín-López, Albert Blanchart, Juan A. De Carlos, Laura López-Mascaraque

**Affiliations:** Department of Molecular, Cellular, and Developmental Neurobiology. Instituto Cajal-CSIC, Madrid, Spain; University of Memphis, United States of America

## Abstract

Dab1 mediates reelin signalling and plays critical roles in early brain development such as the stereotypical positioning of neurons in the brain. The olfactory bulb undergoes a prominent layering reorganization, but shows not apparent differences between wild type and *reeler* in the layer organization. Therefore, an accurate regional and cellular simultaneous analysis of these molecules becomes essential to clarify the role played by Dab1 upon Reelin effect. The present study reveals a strong and consistent Dab1 mRNA and protein expressions, throughout the olfactory bulb layers in both wild type and *reeler* mice. In addition, noteworthy is the pattern of Dab1 location within cell nuclei in both strains. Furthermore, a temporal increment of Dab1 expression levels is detected from P0 to P15 in both strains, being the protein quantity higher in *reeler* than in wild type mice. Altogether, our results revealed that Reln acts directly from projection neurons via the production of different Reln fragments. Changes in the pattern of Dab1 expression could reflect an alternative Reln function in postnatal and adult stages, besides a possible regulation of Dab1 by other molecules distinct to Reln.

## Introduction

Reelin (Reln) is an extracellular glycoprotein secreted by different neuron populations such as Cajal-Retzius cells, granule cells in the cerebellum and mitral and periglomerular cells in the olfactory bulb (OB) [1]–[4]. The role of this molecule involves different processes during development and maturation of CNS as layering [1], [5]–[7], detachment of neuroblasts [8], differentiation of radial cells [9], neurogenesis and gliogenesis processes [10], signaling [11] and dendritic spines development [12]. All these processes required from Reln to bind cell-surface receptors: ApoEr2, VLDL, and α3β1 [13]–[16], and the activation of its intracellular adaptor protein *Disable homolog-1* (Dab1) [17]–[19]. Thus, Reln modulates neuronal cell positioning via Dab1 activation in both developing and mature synaptic networks.

At earlier embryonic stages, Reln and Dab1 mRNA become to be expressed in the forebrain [3], [19], being the Dab1 mRNA regionally detected underneath cortical Cajal-Retzius cells [18], [19]. In the OB, Reln protein is detected in deeper layers and from postnatal day 5 its expression is restricted to mitral and periglomerular cells [3], [11], [20]. Thus, although the distribution and relationship of Reln with its intracellular adaptor Dab1 has been fully addressed [3], [17], [19]–[22], the relationship between the absence of Reln (*reeler* mice) and the presence of Dab1 is currently unknown . There is ample evidence on the layering alterations in the *reeler* OB [8], [23], but the precise localization and changes of Dab1 protein, within the synaptic circuits of the OB, requires further investigation. Thus, we analyzed Dab1 mRNA and protein expression patterns during the early postnatal development of the OB comparing wild type (*wt*) with *reeler* mice. Moreover, using different glial and neuronal markers we characterized the molecular phenotype of Dab1 expressing cells. While immunohistochemical data revealed no differences in Dab1 distribution throughout the OB layers, western blot measurements showed differences between the two strains. Remarkably is the unexpected Dab1 expression in cell nuclei in the OB, suggesting alternative functions of Dab1 protein.

## Materials and Methods

### Animals

Wild type C57 (*wt*) and Reln mutant (*reeler*) mice were obtained from the Cajal Institute mouse breeding facility. All procedures followed the guidelines for animal care of the European Community Council (86/609/CEE) and were approved by the Bioethical Committee at the Spanish National Research Council (CSIC). Animals were deeply anesthetized by hypothermia (P0 and P3) or with i.p. equithesin at lethal dose (P7 and P15) and then transcardially perfused with different solutions according to the experiment. *Reeler* mice were genotyped by PCR.

### Western blot

Anesthetized mice were transcardially perfused with saline, OB were dissected, stripped of meninges and homogenized using a teflon-glass homogenizer (Wheaton Science Products, Millville, USA) in a buffer containing 250 mM sucrose (Merck, Darmstad, DE), 50 mM TrisCl (Roche, Mannheim, DE), 5 mM MgCl_2_ (Panreac, Barcelona, ES), 1 mM 1,4-Dithio-DL-threitol (DTT; Fluka, Steinheimm, DE), 1 mM phenylmethylsulfonyl fluoride (PMSF; Sigma-Aldrich, Steinheimm, DE), 25 µg/ml spermidin (Sigma-Aldrich Co, St. Louis, MO, USA), 1 µg/ml aprotinin (Sigma-Aldrich Co) and 1 µg/ml leupeptin (Sigma-Aldrich Co). Then, tissue was centrifuged (800 g, 15 min) to obtain the crude cytoplasmic fraction (CF) in the supernatant and the nuclear fraction (NF) in the pellet. NF was resuspended in homogenization buffer and centrifuged 700 g to obtain the crude NF. CF and NF protein concentrations were measured by Bradford method using a dye reagent for protein assay (BIO-RAD Laboratories GmbH, München, DE) and then, adjusted at the same quantity with 5X loading buffer (β-mercaptoethanol containing Laemmli buffer). Then, 24 µg of protein were loaded per well in a 7.5% SDS-polyacrylamide gel using a Mini-PROTEAN electrophoresis system (BIO-RAD Laboratories GmbH). Molecular weights (MW) were determined with precision plus protein dual color standards (BIO-RAD Laboratories GmbH). Proteins were transferred to 0.22 µm Protran™ nitrocellulose membranes (Whatman, GE Healthcare) for immunoblotting. Membranes were incubated with phosphate buffered saline (PBS) supplemented with 2% bovine serum albumin (BSA, Sigma-Aldrich Co) to block unspecific binding sites, and overnight incubated at 4°C with specific primary antibodies diluted in PBS-2% BSA (see Table 1). Antibody binding was detected using goat anti-mouse IgG and goat anti-rabbit IgG peroxidase conjugate antibodies (Jackson Immunoresearch, West Grove, PA), diluted 1∶10.000 in PBS for 2 h at room temperature (RT). Bands were visualized using ECL Plus western blotting detection reagents (Amersham GE Healthcare) and exposed to X-ray films (Amersham GE Healthcare). Binding of antibodies was stripped from membranes by Restore PLUS Western Blot Stripping Buffer (Thermo Scientific, Rockford, US). Protein levels were quantified by densitometry of bands, for which X-ray films were digitalized using a GS800 Densitometer (BIO-RAD Laboratories GmbH). OD levels for each sample were subtracted to the background and then Dab1 levels were normalized against load control proteins, β-tubulin and P62 for cytoplasm and nuclei Dab1 levels respectively. Statistical differences between Dab1 OD intensities (n = 3 for each strain and age) were assured applying a One-way and a Factorial ANOVAs respectively, followed by a post-hoc Newman-Keuls test.

**Table 1:** List of antibodies and cell types labelled.

Primary Antibody	Clone	Product Company	Dilution	Antigen
Aconitase	Polyclonal	Sigma-Aldrich	1∶1000	Aconitate-hydratase present in cell mitochondrion
β-Tubulin	5G8	Promega	1∶100.000	Recognize the βIII tubulin protein on neurons
Calbindin (CB)	300	Swant	1∶5000	Localizes D-28k CB which occur in a subset of neurons
Dab1	Polyclonal	Sigma-Aldrich (D1569)	1∶1000	
Dab1	Polyclonal	Chemicon (AB5840)	1∶1000	
DCX	Polyclonal	Cell Signaling	1∶1000	Doublecortin highly expressed in neuronal cells of fetal brain
GAP43	Polyclonal	Chemicon	1∶1000	Growth associated protein 43 highly expressed in neuronal growth cones
GFAP	GA5	Chemicon	1∶1000	Glial fibrillary acidic protein expressed in astrocytes
GFAP	Polyclonal	Dako	1∶1000	Glial fibrillary acidic protein expressed in astrocytes
Histone H1/H5	3h9	Upstate	1∶2000	Linker histone inside of nucleosome structure of the chromosomal fibers
Map2a,b	AP20	Chemicon	1∶1000	Microtubule associated protein-2 expressed in mature neurons
NeuN	A60	Chemicon	1∶1000	Neuronal nuclei protein
Nucleoporin P62	53	BD Biosciences	1∶10.000	A type of nucleoporin of the nuclear pore complex
Parvalbumin (PV)	PARV-19	Sigma-Aldrich	1∶1000	Calcium-binding albumin protein present in GABAergic neurons
RC2	RC2	Hybridoma-Bank	1∶500	A 295 kDa intermediate filament protein present in radial glial cells
Reelin	G10	Chemicon	1∶1000	Detects reelin expressed in a subset of neurons
RIP	NS-1	Chemicon	1∶1000	Detects CNPase in oligodendrocytes and myelin sheats
TH	LNC1	Chemicon	1∶1000	Detects tyrosine hydroxylase enzyme expressed by dopaminergic neurons

### Isolated OB nuclei staining

A portion of the nuclei suspension (see above) was used for intranuclear staining with different markers (Table 1). Nuclei were centrifuged at 800 g and then resuspended in ice-cool 4% paraformaldehyde (PFA) to fix at RT during 15 min. Then, nuclei were washed twice with PBS +0.1% TritonX100 (PBST) and 1 h incubated with rabbit anti-Dab1 at RT. Nuclei were washed twice with PBST and incubated 1 h with the specific secondary antibody Alexa 488 conjugated goat anti-rabbit IgG (Molecular Probes-Invitrogen, Leiden, NL). Nuclei were counterstained with Hoechst (1 µg/ml, Sigma-Aldrich Co). Staining was visualized using a confocal microscope TS5 (Leica).

### In situ hybridization

*Wt* and *reeler* mice (n = 3 for each age and strain) were transcardially perfused with 1 ml/g of ice-cool 4% PFA in 0.1 M diethyl-pyrocarbonate (Sigma-Aldrich Co.) phosphate buffer treated (DEPC-PB). Then, OB were removed and postfixed overnight, cryoprotected in 30% sucrose in DEPC-PB and then sectioned 20 µm thick at the sagittal plane, using a TC1900 cryostat (Leica). In situ hybridization (ISH) was performed using an InsituPro VS device (Intavis Bioanalytical Instruments, AG) with the following protocol: sections were postfixed 10 min at RT with 4% PFA-DEPC, rinsed with PBS, digested 5 min at RT with 1 µg/ml proteinase K (Sigma-Aldrich Co.), fixed again 5 min and acetylated at RT for 10 min. After PBST permeabilization for 30 min, sections were pre-hybridized for 2 h with hybridization solution (1X Denharts, 50% formamide, 250 µg/ml baker yeast RNA, 500 µg/ml herring sperm DNA in 0.2X saline sodium citrate buffer (SSC) at RT. Dab1 probes were hybridized in a hybridization solution containing 350 ng/ml digoxigenin (DIG)-Dab1 RNA probe (preheated to 80°C for 5 min and iced) overnight at 72°C. Unspecified binding sites were blocked for 1 h in 10% NGS (Millipore) supplemented Tris buffered saline (TBS) and then incubated with alkaline peroxidase anti-DIG primary antibody (Roche, Mannheim, DE) at RT overnight. Antibody binding was detected using BM Purple AP substrate precipitating reactive (Roche).

### Immunohistochemistry

Mice (n = 5 labeling for each age and strain) were transcardially perfused, first with heparinized saline solution and then with 1 ml/g of ice-cool 4% PFA. Brains were cryoprotected in 30% sucrose and 20 µm-sectioned at the sagittal plane, using a TC1900 cryostat (Leica). Frozen sections were air dried, permeabilized with PBST and boiled in 10 mM citrate buffer pH 6 to unmask the antigen. Unspecific antibodies binding was blocked with 0.1% PBST supplemented with 10% NGS +0.1% BSA for 1 h at RT and then incubated overnight at 4°C using specific primary antibodies, all diluted in 0.1% PBST +1% NGS (Table 1). Antibody binding was detected with the following secondary antibodies, diluted 1∶1000 in PBST: Alexa 488 goat anti-rabbit IgG and Alexa 568 goat anti-mouse IgG (Molecular Probes-Invitrogen). RC2 antibody was detected by incubating 2 h at RT with biotin conjugated goat anti-mouse IgM (Jackson Immunoresearch) and then incubated 1 h at RT with Alexa 568 conjugated streptavidin (Molecular Probes-Invitrogen). Nuclei were counterstained with Hoechst (1 µg/ml, Sigma-Aldrich Co). Staining was visualized using a confocal microscope TS5 (Leica).

## Results and Discussion

Reln molecule translocates its signal activating phosphorylation of the intracellular adaptor protein Dab1 [24]. These two molecules are closely interrelated, so that one regulates the expression of the other [25], [26]. Thus, Reln-Dab1 interaction is critical regulator of key steps during CNS development, acting in a time-dependent manner in different regions of the brain [19], [20]. Then, regional and cellular quantified distribution of these molecules becomes essential for understanding their role during development. Since the OB is a microcircuitry, with a heterogeneous interneuron population generated throughout development and adulthood, it represents a perfect model to analyze layering and developmental cell maturation in a specific time windows. This led us to address in detail the Reln/Dab1 postnatal expression in *reeler* and *wt* mice and its phenotypical consequences.

### Spatiotemporal expression of Dab1 mRNA and protein in the olfactory bulb

**Figure 1:**
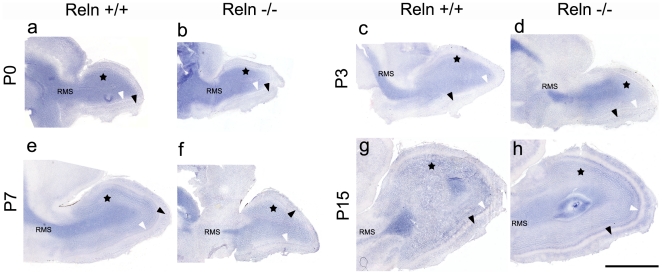
Pattern of Dab1 mRNA location in the postnatal olfactory bulb. Dab1 transcripts are shown in *wt* (a, c, e, g) and *reeler* (b, d, f, h) mice at P0 (a,b), P3, (c, d), P7 (e, f) and P15 (g, h). Transcripts are mainly detected in granular cells (black stars in GcL), mitral cells (white arrows in MCL) and several periglomerular cell populations and intra-glomeruli processes (black arrows in GL). No differences are observed between *wt* and *reeler* mice. Note the high presence of Dab1 mRNA in the rostral migratory stream (RMS) cells. Scale bar: 1 mm.

Localization of Dab1 transcripts was strikingly similar throughout the OB layers in both *wt* and *reeler*. Prominent, Dab1 expression was located in periglomerular and mitral cells, as well as in the granular cell layer (Fig. 1). Although our data revealed Dab1 transcripts in the whole glomeruli from P0 to P7, the expression pattern was similar to those presented in the Allen Brain Atlas [27] (www.brain-map.org).

**Figure 2:**
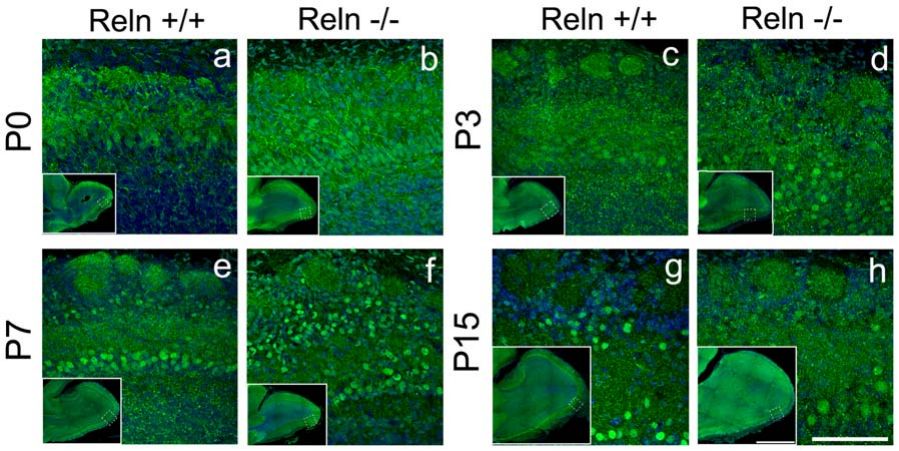
Dab1 protein expression in the postnatal olfactory bulb. Protein expression in wild type (a, c, e, g) and *reeler* (b, d, f, h) mice at P0 (a, b), P3, (c, d), P7 (e, f) and P15 (g, h)**.** Dab1 immunostaining is strongly localized in cell processes of the GL, EPL, and IPL. A dotted pattern is evident in GcL. Mitral and some periglomerular cells are easily identified by a characteristic nuclear Dab1 labeling. Nuclei labeling in periglomerular cells is evident from P3. No marked differences are observed between *wt* and *reeler* at these ages. Nuclei are counterstained with Hoechst (blue). Images represent sagittal sections of OB. Scale bars a-h: 100 µm; inset images: 1 mm.

As occurs with Dab1 transcripts, its protein also showed a homogeneous distribution throughout OB layers in both strains (Fig. 2). These similarities were unexpected, since Dab1 traslocates the Reln signal after is bounded to their receptors. This might represents an alternative role of Dab1 to the Reln pathway, as proposed in the rostral migratory stream cells where Dab1 is triggered by Trombospondin-1 and F-spondin [28], [29]. Other possibilities could be related to the presence of Dab1 isoforms, similar to those phosphorylated independently of Reln in embryonic retinal cells [30], [31] or by mechanisms similar to those underlying the GABAergic interneurons layering [32].

Specifically, Dab1 immunoreactivity was mostly located within three different OB regions (Fig. 2): i) in neuronal processes, primarily in dendrites of the glomerulus compartment (GL), and in both external and internal plexiform layers (EPL and IPL); ii) in cell bodies from both GcL and subependimal zone (SEZ), with an intense labeling in OB radial glial cells which slightly decreases from P0 to P15 (see below); iii) an unpredicted Dab1 expression in cell nuclei of MCL, EPL and GL, as well as in the neuroblasts that reach the OB through the rostral migratory stream. This agrees with the specific nuclear transporting sequences of Dab1 molecule, that facilitates the nuclear translocation describing Dab1 as a nucleocytoplasmic shuttling protein [33]. However, this specific nuclear location of Dab1 protein occurs when the nuclear exporting receptor CRM1 (able for binding to Dab1 sequences) is blocked with leptomycin B [34]. Our data suggest a new nuclear function for Dab1 molecule, since always appeared located in the nucleus, even in the absence of blocking agents for the nuclear exporting receptor CRM1. Moreover, the pattern of a clearly Dab1 nuclear labeling in mitral and periglomerular neurons *in vivo* sections makes possible its use as a specific marker of these cell populations.

**Figure 3:**
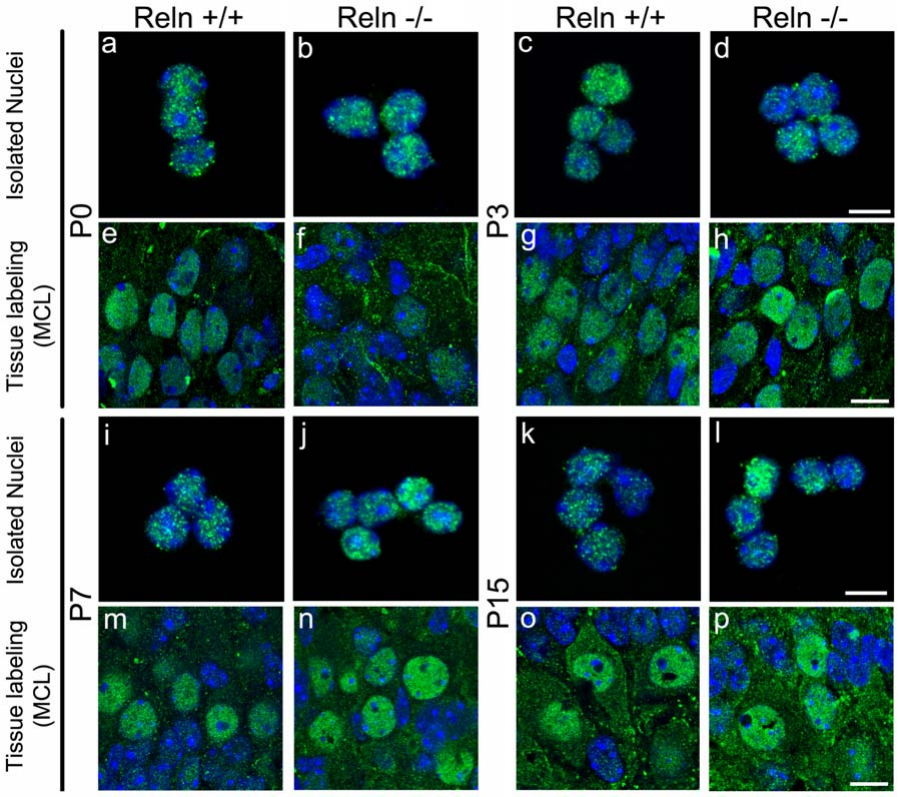
Detection of Dab1 protein within cell nuclei. Labeling of isolated nuclei and tissue sections of the OB in wild type (a, c, e, g, i, k, m, o) and *reeler* (b, d, f, h, j, l, n, p) at P0 (a, b, e, f), P3 (c, d, g, h), P7 (i, j, m, n) and P15 (k, l, o, p). Labeling shows a green dotted pattern in nuclei from both isolated (a–d, i–l) and *in vivo* sections (e–h, m–p). Nuclei are counterstained with Hoechst (blue). *In vivo* images are single confocal sections from MCL (mitral cells layer). Scales bars: 10 µm in e–h, m–p; 5 µm in a–d, i–l.

Because the intriguing immunostaining pattern in the cell nuclei (Fig. 2), we further analyzed this intra-nuclear localization of Dab1 protein on isolated nuclei. As a result, Dab1 protein always appeared clustered in a dotted manner in both *wt* (Fig. 3 a, e, c, g, i, m, k, o) and *reeler* mice (Fig. 3 b, f, d, h, j, n, l, p). Thus, this nuclear pattern (Fig. a–d, i–l) correlated with the *in vivo* labeling (Fig.3 e–h, m–p).

**Figure 4:**
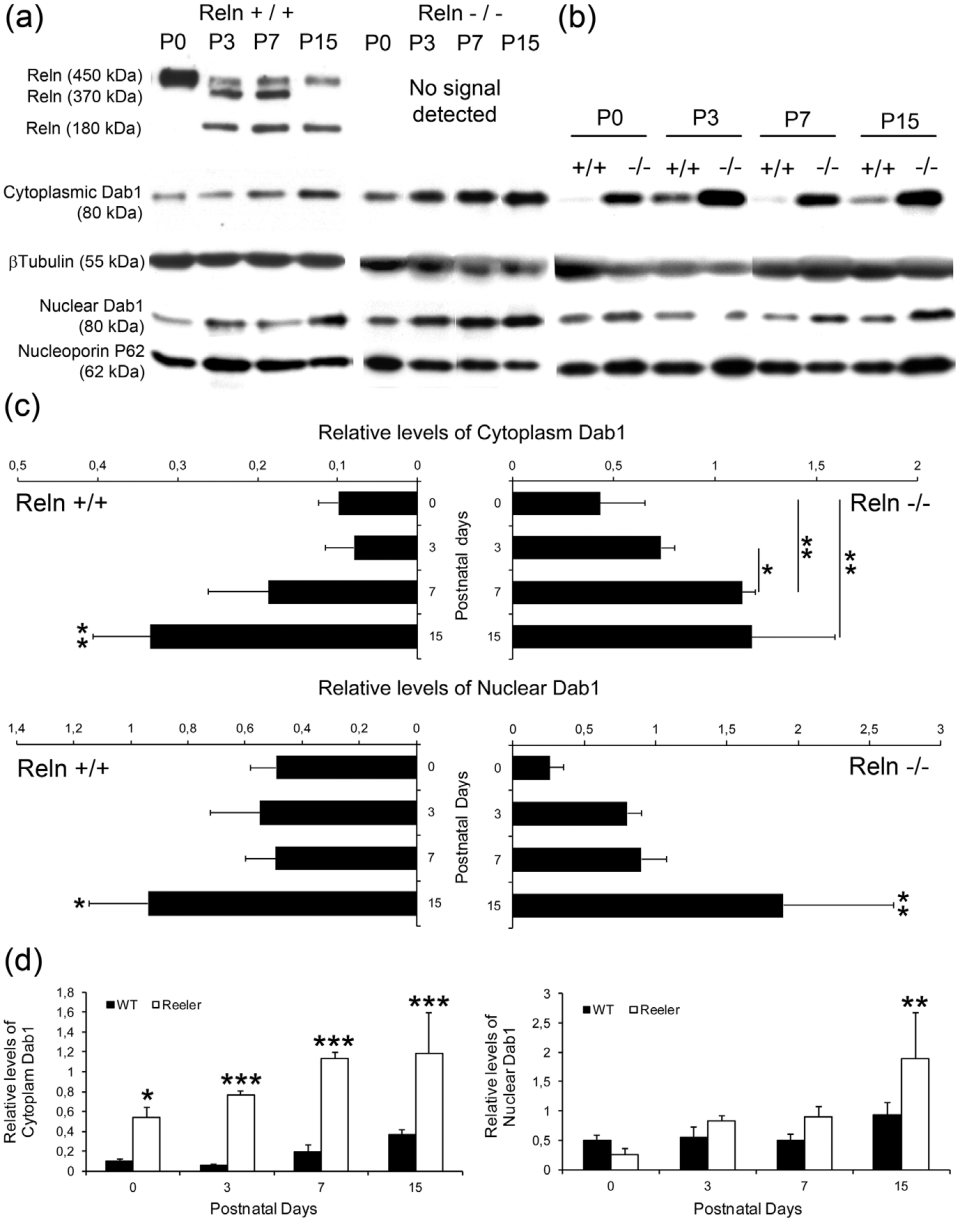
Relative levels of both Reln and Dab1 proteins by western blot in the olfactory bulb. (a) Reln and Dab1 protein bands identified in cytoplasm (CF) and nuclear (NF) fractions of *wt* and *reeler* mice. Identities of protein bands are indicated. Reln is absent in *reeler*. Dab1 quantities are different in CF and NF identified by distinct band intensities. (b) Amount of Dab1 compared between *wt* and *reeler* animals in both subcellular compartments. (c) Comparison of relative levels of Dab1 in CF and NF of *wt* and *reeler* mice. CF amount of Dab1 is statistically higher in *wt* at P15, while in *reeler* the levels are higher at P7 and P15. In NF, Dab1 levels are statistically higher exclusively at P15. (d) Comparison of Dab1 quantities between *wt* and *reeler* animals. Dab1 *reeler* content is significantly higher in CF at all studied ages. In NF this difference is restricted to P15. (* p<0.05; ** p <0.01; *** p <0.001; N = 3)

Quantification of Reln and Dab1 levels were independently performed in both cytosolic (CF) and nuclear (NF) fractions of OB from *wt* and *reeler* tissues. Absence of proteins contamination in tissue extracts was guaranteed by detection of nuclear and cytoplasm proteins in CF and NF samples respectively (Fig. S1a). In addition, cytoplasmic markers (Aconitase and GFAP) were also tested on isolated nuclei (Fig.S1b–g). While aconitase labeling completely avoid cell nuclei (Fig. S1b–d), GFAP shows a labeling circle specifically confined to the nuclear membrane (Fig. S1e–g). This was probably due to the nuclear membrane anchorage [35]. Reln western blot allowed the identification of immunoreactive bands of molecular weights of 450, 370 and 180 kDa (Fig. 4a), corresponding with the full length and two cleavage fragments of the molecule. Full length Reln molecule was detected at P0, the cleavage Reln fragments appeared at P3 and P7, while the 370 kDa fragment was missing at P15. These fragments probably corresponded to a time-course dependent cleavage mechanism, likely produced after C-terminus cleavage by extracellular metalloproteinases [36]. These results suggest that Reln selective fragments might act in a distinct development events via the activation of different metalloproteinases, as occurs for embryonic stages [37]. Further, Reln cleavage would signal cells for a correct positioning in the OB, as suggested for hippocampal granule cells [38].

With respect to cytoplasmic and nuclear Dab1 fractions, protein levels increased from P0 to P15 in both strains (Fig. 4a, c). Statistical analyses of CF-Dab1 levels revealed and increment at P7 and P15 in *reeler,* whereas this increment was restricted to P15 in *wt* (Fig. 4a, c). By other hand, analyses of NF-Dab1 levels showed an increment at P15 in both strains (Fig. 4a, c). The quantity of CF-Dab1 protein was higher in *reeler* than in *wt* in all studied ages, revealing a protein accumulation in OB cells (Fig. 4b, d), while the NF-Dab1 level was just higher in *reeler* at P15. Dab1 increment in both strains strongly suggests an augmented Dab1 function, due to other signaling pathways distinct to the Reln. The higher levels of Dab1 in *reeler* than in *wt* could be due to the requirement of Reln to degrade Dab1 [26], [39]. Thus, in absence of Reln, Dab1 should be accumulated in the cytoplasm, in an unphosphorylated state [16], [19].

To strengthen the Dab1 nuclear and cellular processes localization with the antibody used in this study (from Sigma-Aldrich), we provided supplementary data (Fig. S2) using another anti-Dab1 (from Chemicon). Comparing the labeling of both antibodies, western blot revealed the presence of an 80 kDa band in tissue fractions (Fig. S2a) along with the immunolabeling of isolated nuclei (Fig. S2b). Unexpectedly, cell nuclei and glomerular processes were not labeled in olfactory bulb sections with anti-Dab1 from Chemicon (Fig. S2c). These differences could be related with the immunogens used to produce the antibody, which are different in length. Thus, the immunogen peptide sequence used by Chemicon (aminoacid residues from 400 to 555 of the mouse Dab1 protein, GeneID: 13131; UNIPROT number P97318.2) is longer than that used by Sigma-Aldrich (aminoacid residues from 538 to 555 of the rat Dab1 protein, GeneID: 266729; UNIPROT number Q8CJH2), and contains the total sequence. This could explain the differences in the epitopes recognized by both antibodies. Although both antibodies recognized correctly the Dab1 protein, we suggest that differences in labeling patterns could be related due to both antibodies recognize different epitopes in Dab1 isoforms.

### Molecular characterization of Dab1-expressing cells in the olfactory bulb

**Figure 5:**
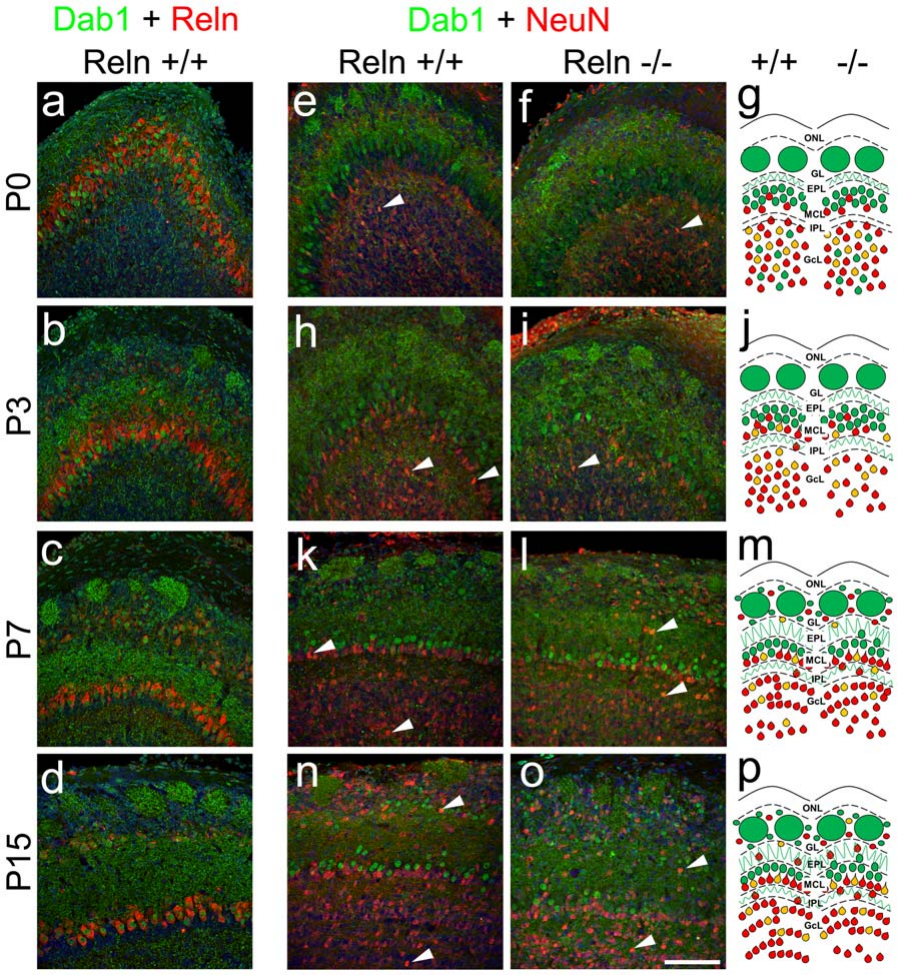
Colocalization of Dab1/Reln and Dab1/NeuN in postnatal olfactory bulb cells. Dab1 (green) and Reln (red) in wild type (a–d); Dab1 and NeuN (red) in wild type (e, h, k, n) and *reeler* (f, i, l, o). Nuclei are counterstained with Hoechst (blue). Images correspond to representative sagittal sections at P0 (a, e, f, g), P3, (b, h, i, j), P7 (c, k, l, m) and P15 (d, n, o, p). Reln shows a complementary location pattern with Dab1 positive nuclei of mitral cells (a–d). At P0, glomeruli are slightly apparent with Reln staining (a). From P3 to P15 glomeruli strongly express Dab1 and periglomerular cells start to express both Reln and nuclear Dab1 (b–d). From P3 the majority of granular and periglomerular cells are positive for NeuN (e–p), many of them with nuclear Dab1 expression (arrows). Cartoons represent the bilayer formation by the mitral cells (nuclear Dab1 staining) with respect to the superficial granular cells (NeuN labeling). This cell positioning is disrupted in *reeler* mice (cartoons). ONL, olfactory nerve layer; GL, glomerular layer; EPL, external plexiform layer; MCL, mitral cells layer; IPL, internal plexiform layer; GcL, granular cells layer; SEZ, subependymal zone. Scale bar: 100 µm.

To analyze the phenotypic profile of Dab1 expressing cells, we performed double immunohistochemistry with different neuronal and glial markers in both strains (Table 2). Nuclei of mitral and some periglomerular cells, identified by Reln labeling at postnatal stages [3], [20], coexpressed Dab1 at all ages (Fig. 5a–d). Moreover, Dab1/NeuN coexpression pattern varied from P0 to P15 (Fig. 5e–p). At P0–P3, double labeled cells were mainly located in the GcL-IPL along with some sparsely labeled cells in the MCL (Fig. 5e–j, arrows). From P7 to P15, Dab1/NeuN cells were also located in EPL and GL (Fig. 5k–p arrows), which could be related to OB layer refinement [40], [41]. This layering process is summarizes in the cartoons (Fig. 5 g, j, m, p). Combining Dab1 nuclear labeling with NeuN marker allows to observe superficial granular cells forming a monolayer just beneath the MCL. This correspond to the granule cells projecting to the most external part of the OB [42], and whose disposition was disrupted in *reeler* [8].

**Table 2:** Extent of Dab1 coexpression with neuronal and glial markers at different postnatal ages in *wt* and *reeler* animals.

Markers	P0–P3	P7	P15
Dab1 / Reln	Mitral (P0) and periglomerular cells (P0–P3)	Mitral and periglomerular cells	
Dab1 / NeuN	MCL, IPL and GcL	None NeuN cell expressed Dab1	
Dab1 / Map2a,b	GL, EPL, MCL, IPL and GcL (P3)	GL, EPL, MCL, IPL and GcL	
Dab1 / RC2	GL, EPL, MCL, IPL, GcL and SEZ	GL, EPL and MCL	None RC2 cell expressed Dab1
Dab1 / CB	No Dab1/CB-PV expressing cell	Dab1 detected in some CB and PV cells	
Dab1 / PV			
Dab1 / TH	Dab1 restricted to nuclei of TH cells	Dab1 in nuclei and process of some TH cells	
Dab1 / GFAP	No Dab1/GFAP expressing cell		
Dab1 / RIP	No Dab1/RIP expressing cell		

**Figure 6:**
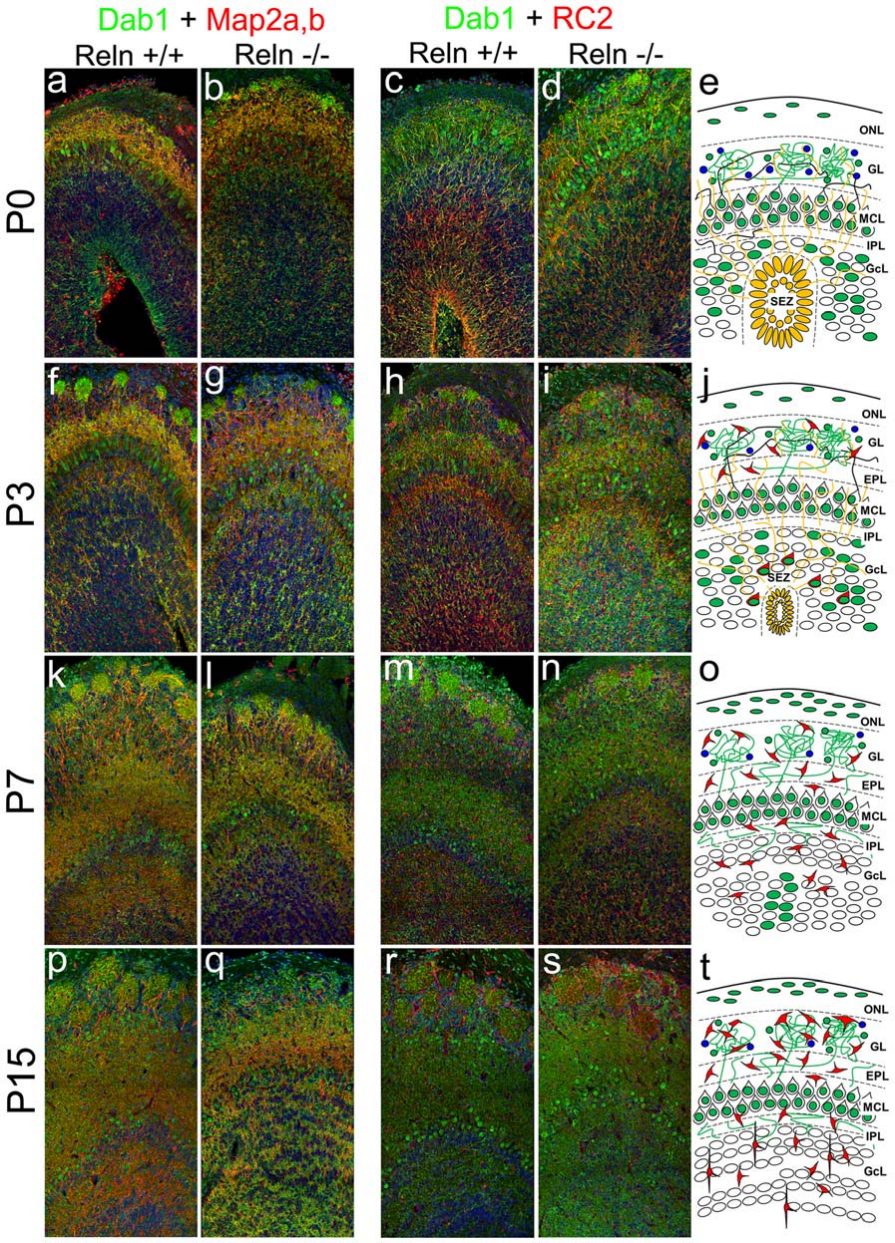
Colocalization of Dab1/Map2a,b and Dab1/RC2 in postnatal olfactory bulb cells. Dab1 (green), Map2a,b (red) (a–b, f–g, k–l, p–q) and the radial glial cell marker RC2 (red) (c–e, h–j, m–o, r–t). OB sagittal sections at P0 (a–e), P3 (f–j), P7 (k–o) and P15 (p–t). At P0 neuronal Dab1 positive processes (Map2a,b) are evident in the GL, EPL and MCL (a, b) while radial glial Dab1 positive processes (RC2) occupied the OB surface (c–e). From P3 onwards, neuronal Dab1 positive neuronal fibers become evident in the IPL and GcL (f–g) while the radial cell processes decreased in quantity. Some Rc2 positive/Dab1 negative cells surround the glomeruli (h–j). Neuronal fibers pattern is similar at P7 (k–i) and P15 (p–q), whereas Rc2 fibers are absent from P7 (m–o). From P7 to P15 all radial glial cells are Dab1 negative (m–o; r–t). Nuclei counterstained with Hoechst (blue). ONL, olfactory nerve layer; GL, glomerular layer; EPL, external plexiform layer; MCL, mitral cells layer; IPL, internal plexiform layer; GcL, granular cells layer; SEZ, subependymal zone. Scale bar: 100 µm.

Coexpression of neuronal marker Map2a,b with Dab1 was observed in both, processes of the GL, EPL and IPL, and in neuronal somas of MCL-GcL at P0–P3, (Fig. 6a–b, f–g). From P7, Map2a,b/Dab1 colocalized in the proximal portions of large processes, mostly located in GL, EPL and IPL (Fig. 6k,l). However, some others Dab1 processes were negative for Map2a,b, but intensively labeled with the radial glial cell marker RC2 (Fig. 6c–e, h–j, m–o, r–t). This marker showed radial glial cell bodies mainly located in the SEZ with thin and large cell processes that extended to the most external OB layers at P0 (Fig. 6). From P3 onwards, RC2/Dab1 positive cells were located in upper OB layers, mostly surrounding the glomeruli, adopting a stellate morphology with shorter cellular processes than those observed at P0. From P7, none RC2 cell expressed Dab1.

**Figure 7:**
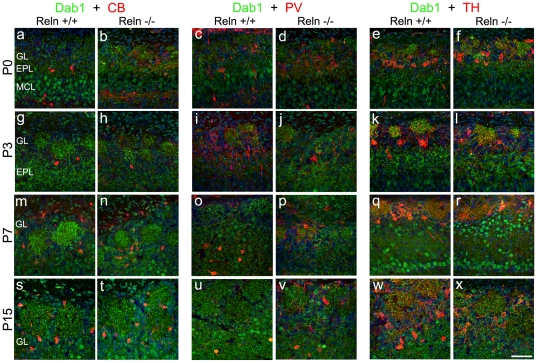
Characterization of periglomerular Dab1 neurons with calbindin (CB), parvalbumin (PV) and tyrosine-hydroxylase (TH) markers. Green color corresponds to Dab1 expression, while the red one corresponds to either CB (a–b, g–h, m–n, s–t), PV (c–d, i––j, o–p, u–v) or TH (e–f, k–l, q–r, w–x) at P0 (a–f), P3 (g–l), P7 (m–r) and P15 (s–x). None CB (a–b) and PV (c–d) positive cells express Dab1 at P0–P3. By contrast, whole TH cell populations express nuclear Dab1 at these ages (e–f, g–i). At P7 some CB and PV are positive for Dab1. By contrary, in *reeler* mice PV cells do not express Dab1 and the cell number is lower (m–p). At this age, TH cells show a lack of Dab1 nuclear expression, which become apparent in some of TH-fibers (q–r). Similar results are observed at P15 (s–x). Nuclei counterstained with Hoechst (blue). GL, glomerular layer; EPL, external plexiform layer; MCL, mitral cell layer. Scale bar: 50 µm.

**Figure 8:**
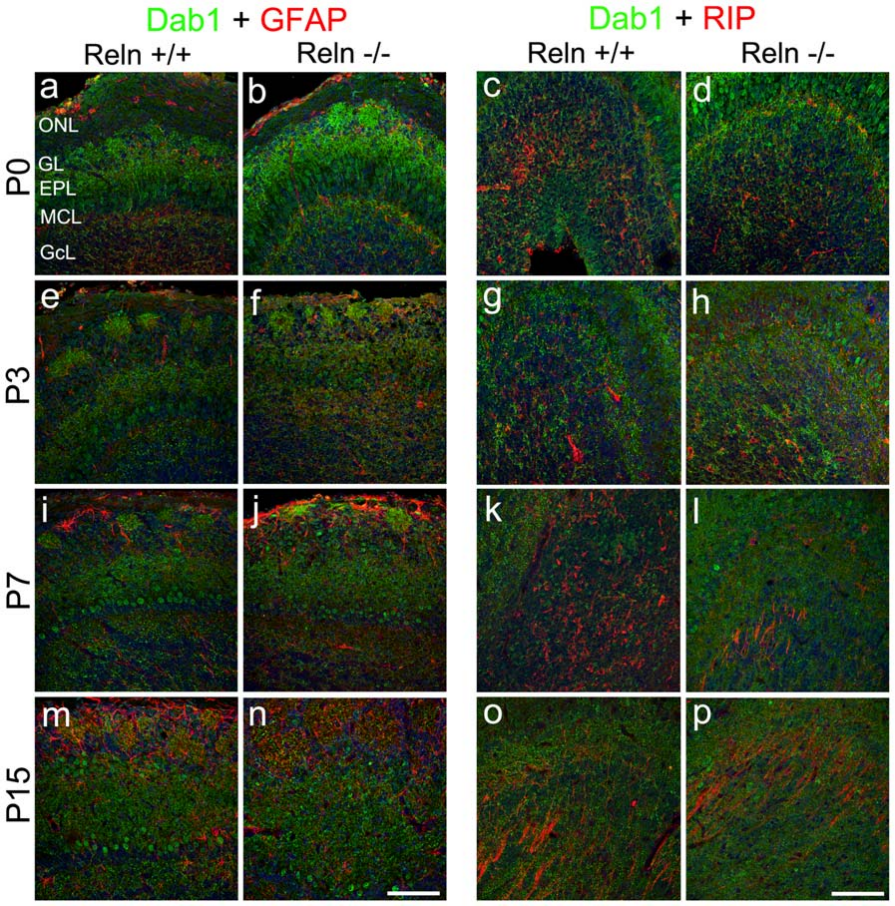
Identification of astroglial and oligodendroglial cell expressing Dab1. P0 (a–d), P3 (e–h), P7 (i–l) and P15 (m–p). Astrocytes are recognized by GFAP (a–b, e–f, i–j, m–n) whereas oligodendrocytes by RIP (c–d, g–h, k–l, o–p). At P0–P3, GFAP (red) is restricted to the ONL and GL (a–b, e–f) and onwards labels the remaining layers, being negative for Dab1 (i–j, m–n). RIP labeling (red) is restricted to non-myelin oligodendrocytes in the GcL at P0–P3 (c–d, g–h). At P7, RIP expression is located in myelin and non-myelin oligodendrocytes (k–l). Non-myelin oligodendrocytes are absent at P15 (o–p). In all cases oligodendrocytes are negatives for Dab1. Labeling pattern is indistinguishable between *wt* and *reeler* animals. Nuclei are counterstained with Hoechst (blue). ONL, olfactory nerve layer; GL, glomerular layer; EPL, external plexiform layer; MCL, mitral cells layer; GcL, granular cells layer. Scale bar: 100 µm.

Specific neuronal markers, calbindin-CB, parvalbumin-PV and tyrosine hydroxylase-TH, identified the neurochemical phenotype of Dab1 positive cells in the glomeruli in both genetic backgrounds. Dab1 colocalized with CB and PV cells from P7 onwards (Fig. 7a–d, g–j, m–p, s–v), being negative in earlier ages. Dab1/TH coexpression was restricted to the nucleus at P0–P3 while at P7 the colabeling was detected in some TH-positive processes inside the glomeruli (Fig. 7e–f, k–l, q–r, w–x). None glial marker (GFAP, RIP) colocalized with Dab1 cells in both *wt* and *reeler* mice (Fig. 8).

In summary, our data revealed that Reln, secreted by mitral and periglomerular cells, suffered a cleavage process during the postnatal OB development probably related to cell lamination. Its main adaptor protein, Dab1, showed an undistinguishable transcript and protein expression patterns between *wt* and *reeler* mice. This suggests alternative roles in the Dab1 function not linked to Reln during the OB postnatal development. This idea is reinforced by the higher quantity of Dab1 in *reeler* respect to *wt*. Additionally, the increment in Dab1, from P0 to P15, suggests further roles of this molecule in the OB postnatal development. Lastly, the unexpected presence of Dab1 labeling in cell nuclei is probably linked to alternative Dab1 functions in the nuclear activity.

## Supporting Information

Figure S1 **Control western blot to test protein contamination in tissue fractions.** (a) Immunodetection of cytoplasm and nuclear proteins in NF and CF respectively in OB extracts from both *wt* and *reeler* mice. (b) Immunohistochemistry on isolated nuclei with cytoplasm proteins at P0 (b, e), P7 (c, f) and P15 (d, g). By western blot are detected bands of nucleoporin P62 in the CF and a slight labeling of both aconitase and GFAP in the NF (a). Aconitase marker is completely absent in isolated nuclei (b–d), whereas GFAP labeling appears delimiting the nuclear membranes (e–g), Scale bar: 5 µm.
(TIF)

Figure S2 **Dab1 expression using two different anti-Dab1 antibodies by western blot and immunohistochemistry.** (a) Western blot of *wt* OB extracts using two anti-Dab1 antibodies from Chemicon and Sigma-Aldrich. Both labeled the specific 80 kDa band correspond to Dab1 protein. Antibody from Chemicon is not being able to detect the increase in protein levels from P0 to P15 as detected by Sigma-Aldrich antibody. (b) Labeling of isolated nuclei with both anti-Dab1 antibodies show a similar dotted pattern inside the nuclei at P0, P7 and P15. (c) Labeling of OB sagittal sections using the anti-Dab1 from Chemicon. In this case the labeling is broadly similar to that observed with the Sigma-Aldrich antibody, which is mainly found in periglomerular cells, MCL and in GcL. A difference respect to the Sigma-Aldrich antibody is the absence of nuclear and cell processes labeling with the Chemicon antibody. Scale bars: 100 µm and 1 mm in the inserts.
(TIF)
