# Supplementary figures and images for: Dab1 (Disable Homolog-1) Reelin Adaptor Protein Is Overexpressed in the Olfactory Bulb at Early Postnatal Stages
Source: PLoS One. 2011 Oct 25;6(10):e26673. doi: 10.1371/journal.pone.0026673 (PMC3201967; doi:10.1371/journal.pone.0026673)

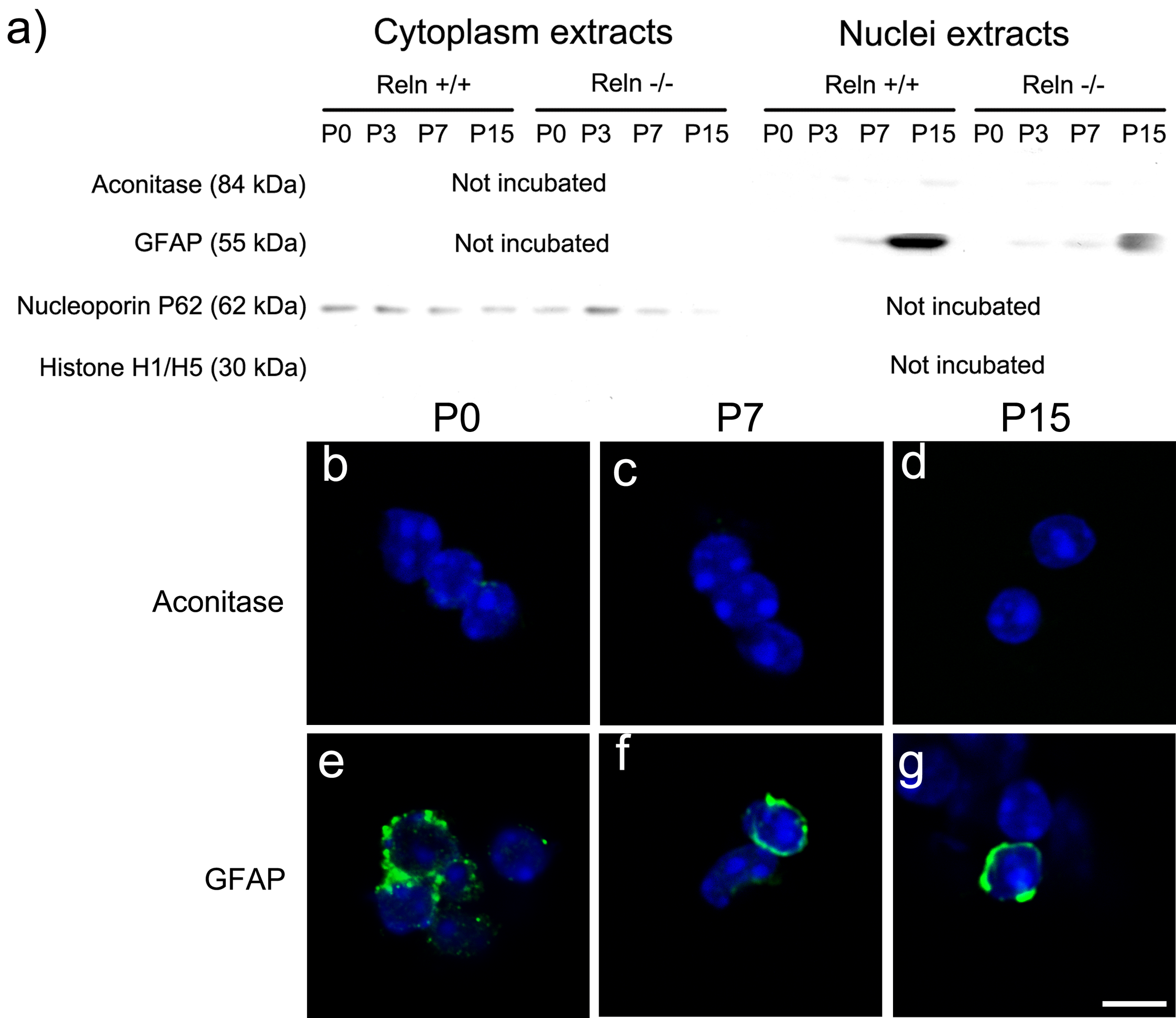

Supplement: Figure S1 — Control western blot to test protein contamination in tissue fractions. (a) Immunodetection of cytoplasm and nuclear proteins in NF and CF respectively in OB extracts from both wt and reeler mice. (b) Immunohistochemistry on isolated nuclei with cytoplasm proteins at P0 (b, e), P7 (c, f) and P15 (d, g). By western blot are detected bands of nucleoporin P62 in the CF and a slight labeling of both aconitase and GFAP in the NF (a). Aconitase marker is completely absent in isolated nuclei (b–d), whereas GFAP labeling appears delimiting the nuclear membranes (e–g), Scale bar: 5 µm. (TIF) [file pone.0026673.s001.tif]

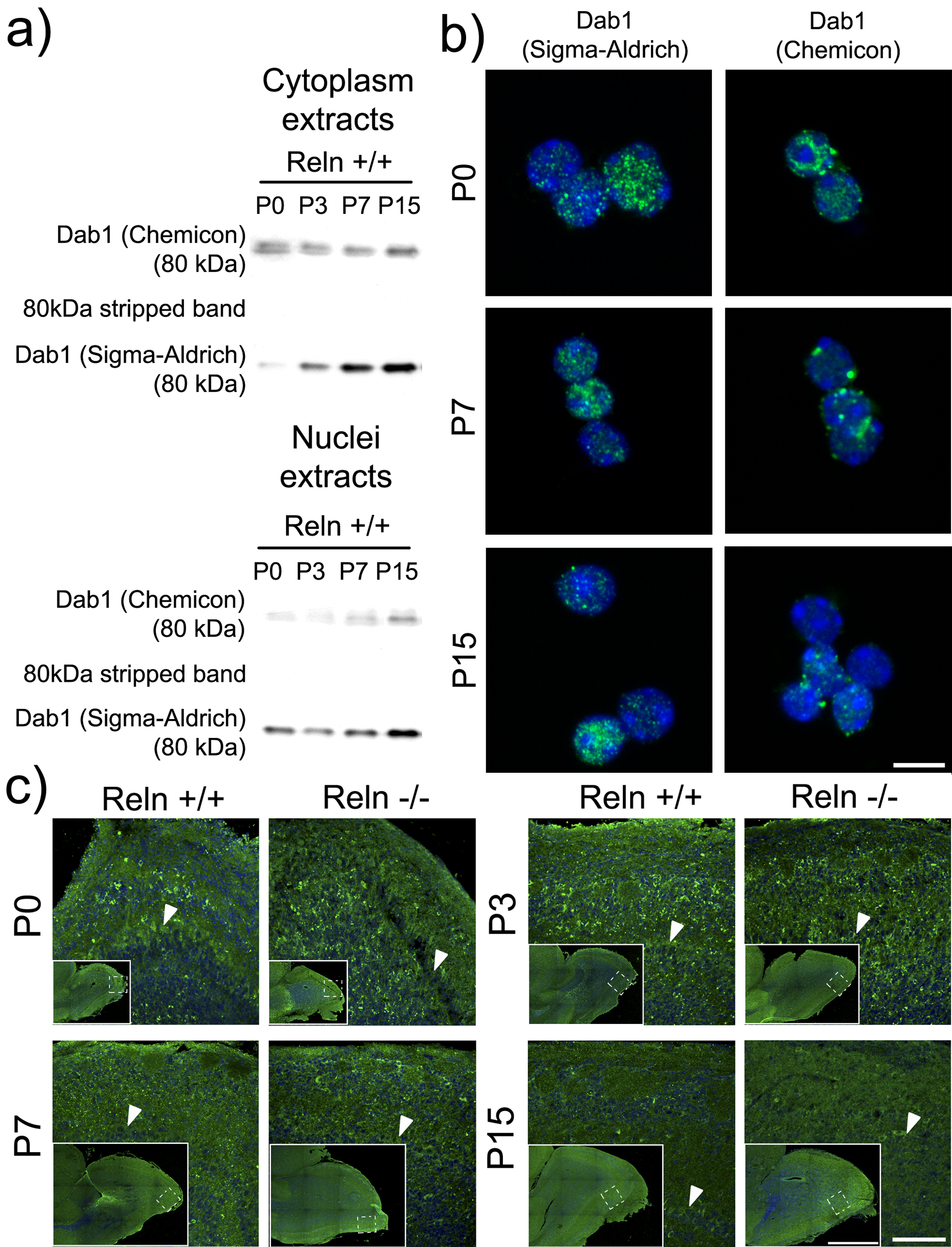

Supplement: Figure S2 — Dab1 expression using two different anti-Dab1 antibodies by western blot and immunohistochemistry. (a) Western blot of wt OB extracts using two anti-Dab1 antibodies from Chemicon and Sigma-Aldrich. Both labeled the specific 80 kDa band correspond to Dab1 protein. Antibody from Chemicon is not being able to detect the increase in protein levels from P0 to P15 as detected by Sigma-Aldrich antibody. (b) Labeling of isolated nuclei with both anti-Dab1 antibodies show a similar dotted pattern inside the nuclei at P0, P7 and P15. (c) Labeling of OB sagittal sections using the anti-Dab1 from Chemicon. In this case the labeling is broadly similar to that observed with the Sigma-Aldrich antibody, which is mainly found in periglomerular cells, MCL and in GcL. A difference respect to the Sigma-Aldrich antibody is the absence of nuclear and cell processes labeling with the Chemicon antibody. Scale bars: 100 µm and 1 mm in the inserts. (TIF) [file pone.0026673.s002.tif]
